# Strigolactones in Plants and Their Interaction with the Ecological Microbiome in Response to Abiotic Stress

**DOI:** 10.3390/plants11243499

**Published:** 2022-12-13

**Authors:** Sabry Soliman, Yi Wang, Zhenhai Han, Tariq Pervaiz, Ashraf El-kereamy

**Affiliations:** 1 Department of Botany and Plant Sciences, University of California Riverside, Riverside, CA 92521, USA; 2 Department of Horticulture, Faculty of Agriculture, Ain Shams University, Cairo 11566, Egypt; 3 Department of Fruit Science, College of Horticulture, China Agriculture University, Beijing 100083, China

**Keywords:** strigolactones, abiotic stress, ecological microbiome, phytohormones

## Abstract

Phytohormones play an essential role in enhancing plant tolerance by responding to abiotic stresses, such as nutrient deficiency, drought, high temperature, and light stress. Strigolactones (SLs) are carotenoid derivatives that occur naturally in plants and are defined as novel phytohormones that regulate plant metabolism, growth, and development. Strigolactone assists plants in the acquisition of defensive characteristics against drought stress by initiating physiological responses and mediating the interaction with soil microorganisms. Nutrient deficiency is an important abiotic stress factor, hence, plants perform many strategies to survive against nutrient deficiency, such as enhancing the efficiency of nutrient uptake and forming beneficial relationships with microorganisms. Strigolactone attracts various microorganisms and provides the roots with essential elements, including nitrogen and phosphorus. Among these advantageous microorganisms are arbuscular mycorrhiza fungi (AMF), which regulate plant metabolic activities through phosphorus providing in roots. Bacterial nodulations are also nitrogen-fixing microorganisms found in plant roots. This symbiotic relationship is maintained as the plant provides organic molecules, produced in the leaves, that the bacteria could otherwise not independently generate. Related stresses, such as light stress and high-temperature stress, could be affected directly or indirectly by strigolactone. However, the messengers of these processes are unknown. The most prominent connector messengers have been identified upon the discovery of SLs and the understanding of their hormonal effect. In addition to attracting microorganisms, these groups of phytohormones affect photosynthesis, bridge other phytohormones, induce metabolic compounds. In this article, we highlighted the brief information available on SLs as a phytohormone group regarding their common related effects. In addition, we reviewed the status and described the application of SLs and plant response to abiotic stresses. This allowed us to comprehend plants’ communication with the ecological microbiome as well as the strategies plants use to survive under various stresses. Furthermore, we identify and classify the SLs that play a role in stress resistance since many ecological microbiomes are unexplained.

## 1. Introduction

Abiotic stresses are the most significant limiting factors of plant survival and growth under the increasing crisis of climatic changes. Numerous studies tried conducted to find a solution for different plant species to cope with various stressors. Plant hormones (phytohormones) are organic molecules that cause signaling effects in plant tissues. Cell elongation, phototropism, stress tolerance, apical dominance, plant growth improvement, senescence, and dormancy are a few of many processes considered as physiological functions of phytohormones. The impacts on plants are substantial despite the minimal concentration of secretion and significantly improve plant tolerance against abiotic stress [1]. In order to adapt to adverse conditions, plants have developed various responses by evoking several signals that cause metabolic and genetic pathways to be reprogrammed [2,3].

Strigolactones (SLs), was identified as plant hormones that play regulatory roles against abiotic stresses in plants, due to their essential role in regulating plant growth and development [4,5]. The first naturally occurring germination stimulant for Striga was isolated as early as 1966 from root exudates of cotton (*Gossypium hirsutum* L.), which is neither a host for Striga nor Orobanche [6,7,8]. SLs are a class of terpenoid-derived compounds that were first discovered as (+)-strigol, which stimulates seeds germination from the parasitic plant Striga [6]. Many researchers reported strigolactone (SL) as a newly identified phytohormone [9,10,11]. Strigolactones improve control the development patterns and interactions between nearby colonies in moss [12,13] Furthermore, SLs participate in metabolic processes acting against biotic and abiotic stress [4,14,15]. The production of SLs in plants is strongly controlled and influenced by the various kinds of stressors that they experience at different growth phases. Recent evidence of interactions between SLs and other phytohormones, such as abscisic acid, in plant responses to abiotic stressors, implies that SLs actively engage in phytohormone-controlled regulatory networks of plant stress adaption [16].

Several studies were focused on SLs since they strongly demonstrate many internal and external responses to plant growth and development [5,8,17]. They regulate lateral roots and root hairs, gravitropism, soil microbes, vasculature development, nutrient and photoassimilate capture and allocation, light responses, leaf shape, leaf senescence, drought, and salinity tolerance [18]. Crosstalk with other known hormones was discussed by several investigations, and the signaling between SLs and other plant hormones may demonstrate their physiological function. Plants regulate their growth by simultaneously sensing and responding to both the external environmental signals and the internal developmental signals [1,4]. For instance, plant phytohormones may have responses to the signaling pathways of photoreceptor phytochromes for improving the photosynthesis processes. The phytohormones also affect photoreceptor signal transduction at cellular levels [18]. The crosstalk of strigolactone with other phytohormones is widely investigated. Phenomena caused by these interactions, such as defense against abiotic stress and attraction of microorganisms, have been explored. Plant morphological adaptability in response to changes in environmental factors is largely influenced by phytohormones. Strigolactones (SLs) are carotenoid-derived hormones that affect various aspects of development and interaction with microorganisms. They have been proposed as mediators of environmental stimuli in resource allocation processes; as a result, their pathways must be responsive to environmental cues in order to contribute to adaptive adjustments [19,20]. The research studies indicate that plants make efficient use of their secondary metabolites to defend themselves and make their environment suitable. For instance, plants actively release volatile compounds to repel herbivores and, at the same time, to draw in natural enemies that are specifically adapted to fight the herbivores [21,22].

There is a global demand for compounds that can help plants to gain tolerance against abiotic stresses such as drought, high-temperature stress, and nutrient deficiency or starvation. SLs alleviate the impact of nutrient deficiency, especially phosphorus and nitrogen, on the plant [23,24,25,26,27,28,29]. In this article, we reviewed the significance of strigolactone, its functions, and research gaps in order to investigate the botanical responses in various plant species to stressors.

## 2. Strigolactone Biosynthesis, and Signaling Pathways

### 2.1. Biosynthesis Pathway

**Figure 1:**
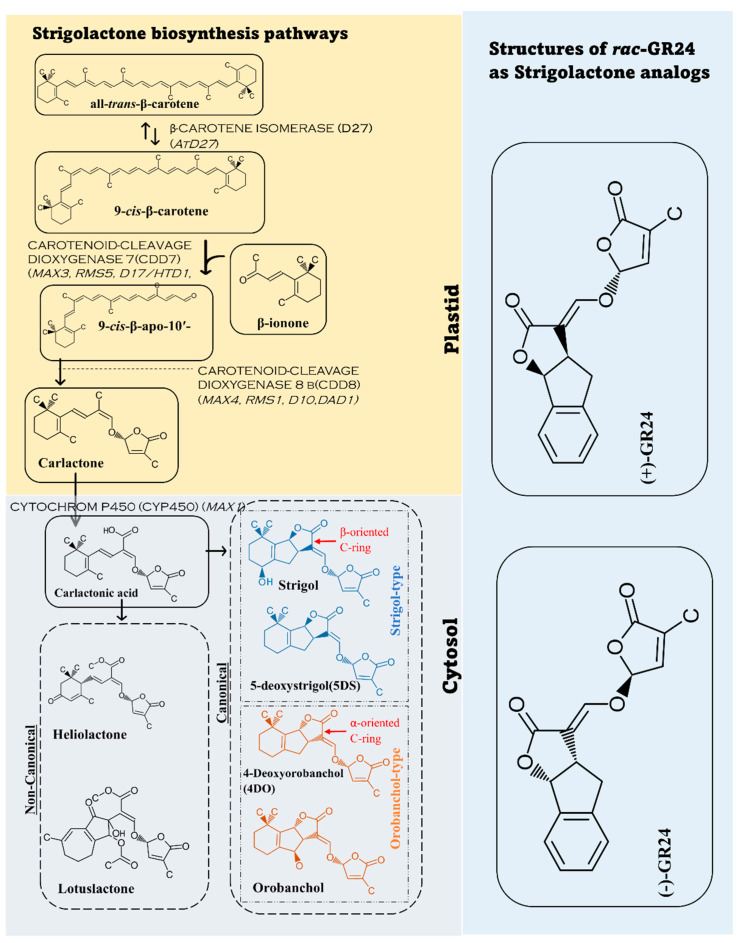
Strigolactone biosynthesis from carotenoids. In plastids all–*trans*–β–carotene goes through different enzymatic reactions to carlactone. Then, the carlactone transforms to form carlactonic acid in the cytosol (cytoplasm) to form various forms of strigolactones. The figure demonstrates two common examples of SLs in non-canonical form, four common ones of canonical form and the most common strigolactone analog, GR24.

Biosynthesis of the strigolactone molecules and SL-like compounds is carried out by β-CAROTENE ISOMERASE (D27) and a group of other enzymes known as CAROTENOID-CLEAVAGE DIOXYGENASE (CCDs group). There are two main dioxygenases groups including CCD7 and CCD8 [30]. In the biosynthetic pathway, D27 has 2 ways of dissociation by converting all-*trans*-β-carotene to 9-*cis*-β-carotene and vice versa. 9-*cis*-β- carotene is converted to 9-*cis*-β-apo-10′carotenal as CCD7 attacks the 9′ and 10′ bonds of β -carotene, and CCD8 cleaves 9-*cis*-β-apo-10′carotene and forms carlactone. Carlactone is transformed into carlactonic acid (CLA) in the cytoplasm by sub family CYTOCHROM P450 (CYP450). Carlactonic acid leads to the formation of 5-deoxystrigol(5DS),4-deoxyorobanchol(4DO), and other forms of SLs [31,32,33]. Although the reactions of converting all-trans-β-carotene happen in plastids, the formation of strigolactones are carried out in the cytoplasm. This happens when carlactone (CL), the precursor of strigolactones, is transported to the cytoplasm where strigolactone and SL-like compounds are formed [31]. Different plants have different enzyme members from the families we mentioned. [15,23,33,34,35]. Many synthetic analogs such as GR24 have been developed and studied. Further studies about synthetic analogs and their characteristics and effects need to occur. The biosynthetic pathway and the common analogs GR24 are explained in Figure 1 [23,34,35,36,37]. 

### 2.2. Signaling Pathways

#### 2.2.1. Impact on Branching and Leaf-Stem Angle with Relatioship to Gravitropism

**Figure 2:**
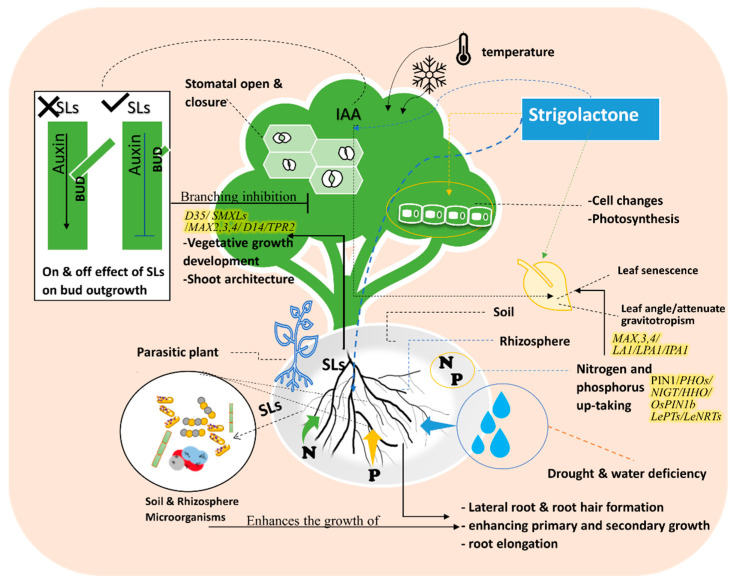
Effects of Strigolactone analogs (e.g., GR24) on the plant’s vegetative growth and signaling pathway under abiotic stress in relation to plant biological characteristics and soil & rhizosphere microbiome. The figure shows the role of SLs on nitrogen and phosphorus uptake. Root and Shoot architecture changes identify the effects of SLs in modifying the plant structure to cope with the abiotic stress. The figure demonstrates that SLs impact shoot branching. The plant utilizes SLs to preform mechanisms that defend itself against different abiotic stresses such as drought, senescence, temperature, light, and nutrient deficiency.

D53-like SMXLs regulate leaf morphology and SL-induced SMXL6 degradation requires *D14* and *MAX2* [38,39]. D53-like SMXLs interact with *MAX2* and *D14*. D53-like SMXLs interact with *TPR2* and exhibit transcriptional repression [39]. It is believed that D53 regulates the expression of genes essential for the development of secondary shoots in the nucleus [40]. In the signaling activation of branching, auxin regulates “*MORE AXILLARY GROWTH” MAX3* and *MAX4* gene expression, these two genes are also influenced by strigolactone during branching inhibition control [41]. *MAX3* and *MAX4* participate in shoot branching and architecture signaling through the gravitropism isolation of *LAZY1(LA1)* suppressors, which revealed the involvement of SLs in shoot gravitropism/rice tiller angle Figure 2. SLs attenuate shoot gravitropic response in rice by modifying trilling angles. SL regulation of rice shoot gravitropism is dependent on indigenous auxin levels [42]. SL-mediated shoot gravitropism is conserved in Arabidopsis and many other plant species. For instance, it was reported in rice that SLs is a moderator of some genes such as *LAZY1(LA1), LOOSE PLANT ARCHITECTURE1(LPA1),* and *IDEAL PLANT ARCHITECTURE1 (IPA1)*, which are involved in shoot branching inhibition and shoot gravitropism. Thus, SLs regulate tiller/branch angle in different plant species, indicating that shoot gravitropism is the key component dictating the proper positioning of shoot branches (Figure 2) [42,43].

#### 2.2.2. Impact on Low-Light Stress

GR24 application increased the activity and gene expression of antioxidant enzymes, and it reduced malonaldehyde (MDA) and hydrogen peroxide (H_2_O_2_) content in Low Light stressed plants (LL-stressed plants) [44,45]. These results suggested that exogenous application of GR24 enhances plant tolerance to LL and improving photosynthesis by promoting utilization of light energy to alleviate photosystem injuries induced by excess light energy and ROS as well as enhancing photosynthesis efficiency to improve plant growth [45,46]. Exogenous GR24 application on tomato seedlings reduces the negative effects of low light exposure in at least three ways: by reducing growth inhibition, improving photosynthetic efficiency, and relieving oxidative stress [45,47,48]. In addition, the application of GR24 effectively alleviates the photoinhibition of photosystems I and II (PSII and PSI) under high light stress mainly by balancing excitation energy and promoting the electron transfer chain between two photosystems, thus enhancing CEF, PQ pools, and quantum yield of PSII and PSI photochemistry [45]. Furthermore, plants growing in LL exhibit reduced levels of the enzymes photosystem II (PS II), ATP synthase, cytochrome b/f, and ribulose-1,5-bisphosphate carboxylase/oxygenase (Rubisco), as well as poorer electron transport (ETR) and CO2 consumption [45,49].

#### 2.2.3. Impact on Nitrogen and Phosphorus Deficiency

**Figure 3:**
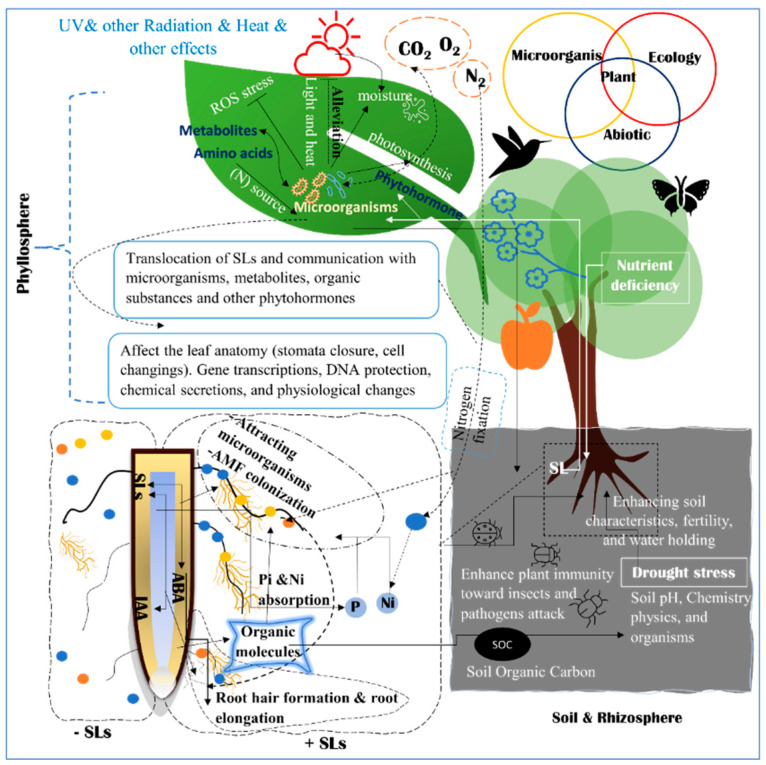
The complex relations between plants, ecology, and microorganisms. The rhizosphere, soil, and root system interact together to alleviate or eliminate the effects of abiotic stress. The phyllosphere ecosystem in the top portion of the figure has a different microbiome. Phyllosphere is composed of all parts above the ground. There are different mechanisms to alleviate abiotic stress with the cooperation of the surrounding ecosystems, physiological processes, and participation of phytohormones and organic substances. The relationship between plants and organisms in the rhizosphere, phyllosphere, and endosphere is demonstrated.

SLs consider the modulating expression factor of regulatory genes as a signaling pathway for nitrogen (N) and phosphorus (P) starvation defense mechanisms [50]. Therefore, many regulatory genes are involved in N and P regulations as well as signaling pathways of N–P integrators *PHOs* family [50,51,52]. SLs also regulate *NIGT/HHO* involved in the phosphorus deficiency signaling pathway [50]. *LePTs* and *LeNRTs* families are responsible for improving phosphorus and nitrogen absorption efficiency [50,52,53,54]. A signaling impact of D3-dependent and SLs biosynthesis in the suppression of tiller bud outgrowth under Pi deficit was identified in several SL signaling pathways [26,53]. *OsPIN1b* responds to low levels of N and P and regulates the activities in the root apical meristem, which leads to the rice seminal root elongation (Figure 2 and Figure 3) [55].

#### 2.2.4. Impact on Other Pathways

Moreover, the expression of several *CAB* genes induced by auxin-SLs may increase the activation of photosynthesis. Several auxin-activated metabolic pathways may be decreased by GR24 as SLs analog. A series of downstream auxin genes are utilized by SLs to alter the tomatoes’ response to auxin. Simultaneously, the biosynthesis of SLs is regulated by auxin using different genes in the Carotenoid biosynthesis pathway [56]. Strigolactone is produced in the roots but can be transported to the shoots [57]. By investigating different *PIN1* trafficking dynamics in roots compared to shoots, it was found that strigolactone-triggered PIN1 PM depletion carries a more significant effect in the shoot as opposed to the root [58].The chemical stability of SLs depends on experimental conditions such as the solvent, pH, and the presence of nucleophiles. SLs are stable in root exudates which are usually composed of an oily mixture of various chemicals that play a crucial role in the plant’s defense against pathogenic attacks. In contrast, SLs exhibit limited stability in aqueous solutions and degrade when removed via several extraction and chromatographic steps [59]. These synthetic analogs are more stable, but they are less active than their natural counterparts. One of the most potent and commonly used SL analogs is GR24, a synthetic analog of strigol synthesized by Gerald Rosebery [59,60]. 

SLs contain several stereogenic (chiral) centers; for example, strigol contains three stereogenic (chiral) centers, resulting in eight possible stereoisomers [61]. At present, two main families of natural SLs are known, namely the strigol family and the orobanchol family, with (+)-strigol and (−)-orobanchol BC stereochemistry, respectively. Natural SLs have a rather complex structure, therefore synthesizing them involves several stages. For instance, enantiopure (+)-strigol must be synthesized in at least 20 stages [59].

Since strigolactone is transported in two separate pathways, from shoot to root and vice versa, further experimental study investigations are required to better understand how this substance is distributed inside plants and transported via various tissues. Transportation from the root as the source of strigolactone showed that high concentrations of synthetic strigolactone increase the export of SLs from the root [8].

## 3. Phytohormones Crosstalk with Strigolactones for Defending against Abiotic Stress 

**Figure 4:**
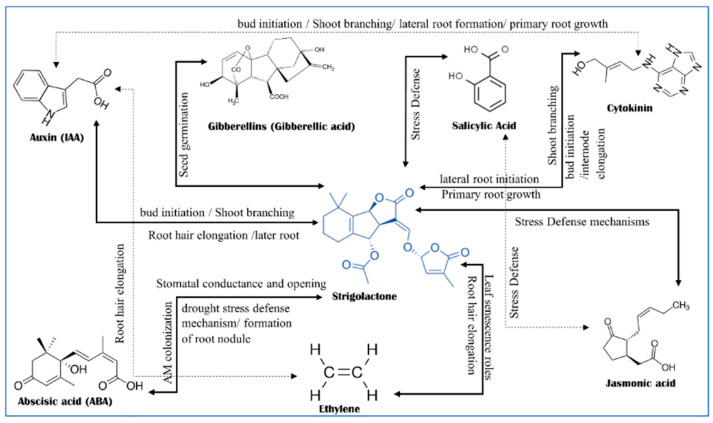
The crosstalk between Strigolactones and other phytohormones and the consequences of growth development. Many physiological and morphological changes occurred as a result of the communications between SLs and other phytohormones.

Plant hormones, such as auxin, cytokinin, gibberellin, abscisic acid, ethylene, salicylic acid, and jasmonate, are a group of Phyto-chemical compounds that act at very low concentrations in plants. There are also peptide hormones and nucleic acids which produce various chemical signals that act as hormones [18,62]. Strigolactones (SLs) are involved in multiple physiological activities, metabolisms, and responses including root formation and architecture, inhibition of shoot branching and promotion of leaf senescence as mentioned in Figure 4. Moreover, strigolactones directly protect plants from biological and abiotic stressors. Chemical regulation techniques are excellent horticultural approaches for increasing stress tolerance [2,7,33,37]. SL, having hormonal functions, crosstalks with other phytohormones (Figure 4) [18,63]. SL’s mode of action varies in different plant organs during cell metabolic activities. Branching inhibition is the most significant function. Interactions with symbiotic arbuscular mycorrhizal (AM) fungi induce hyphal branching, which is related to the crosstalk between SLs and ABA [57,62,64,65,66,67]. Auxin, cytokinin, and SLs have different hormonal influences, crosstalk, and effects [68]. Auxin promotes plant apical dominance by inhibiting the messenger transportation through the stem for bud signaling or initiation. Strigolactone blocks the effects of auxin by stimulating bud outgrowth, thus strigolactone application prevents bud outgrowth (Figure 2, Figure 3 and Figure 4) [23,62,63,68,69,70]. 

SLs are involved in hormonal interactions in root growth and development, including branding effects. With hormonal crosstalk with auxin, SLs pause the apical dominance effect and terminate the axillary bud dormancy. The scientific fact is that when axillary buds are removed or injured, auxin transfers from the active axillary buds and moves through the stem tissues to induce the initiation of lateral buds while raising the concentration of auxin [71,72,73,74,75,76]. The main apparent factor is the inhibition of shoot branching interactions with symbiotic arbuscular mycorrhizal (AM) fungi as it can induce hyphal branching [65]. SLs act as auxin transporters while simultaneously inhibiting lateral bud growth [62,68], and subsequent application of SLs enhances apical dominance. While N-1-naphthylphthalamic acid (NPA) has a significant response to bud outgrowth, it harmonizes the effects among auxin, SLs, and cytokinins toward organizing the bud’s outgrowth and inhibition. 

The interaction of the SLs and abscisic acid (ABA) signaling pathways in *A. thaliana* plays an essential role in regulating stomatal development and function under drought stress [17,62,63]. Contrary to SLs, Cytokinin (CK) regulates plant growth in a way that favors shoot development but is antagonistic to root development and drought tolerance responses [4]. Crosstalk exists between the SLs and ABA regulating stomatal closure as well [63,77]. There are interactions among SLs, CKs, and ABA, as responding to different abiotic stresses such as drought stress [4] and nutrient deficiencies [78]. The physiological processes reacting against these abnormal conditions may upregulate and downregulate the photosynthetic output substances. Chlorophyll content and different secondary metabolite changes have been noticed as an interactive response between various hormones and strigolactone. This strengthens the plant and allows it to cope with adverse conditions. 

Interactions among SLs, jasmonic acid (JA), and salicylic acid (SA) act in defense against several abiotic stresses [62,63]. Fungal and microbial infections could also be affected by the combination of SL-SA. This relation could be a crosstalk between each other’s and against the pathogenic effects of microorganisms [14,79,80,81]. Ethylene and strigolactone play necessary roles in plant growth and development, the response against plant pathogens, root elongation and growth, and senescence [62,63,82]. Strigolactones play a significant role in preventing unnecessary leaf senescence in temporary plant stress. Thus, there is a crosstalk between strigolactone and ethylene to alleviate leaf senescence [83]. Gibberellic acid (GA) showed relations with Sls in seed germination and hypocotyl elongation [62]. The crosstalk between SLs and the other hormones as responding to a vital physiological process in the trees and perennial crops (e.g., Fruit trees) still needs more study for identification. Figure 4 shows some crosstalk responses for a definite process. 

## 4. Exogenous Application of Strigolactone for Improving Some Plant Phenotypic Characteristics

Many researchers have studied the natural exudation of strigolactones endogenously and have found that carotenoid precursors may synthesize SLs as a secondary metabolite in the roots [15,34,35]. G24 is one of the SL analogs (Figure 1) often used for its effects on different stress and physiological processes It is also used to change the phenotype of plants to a non-branching trait. SLs are hormones and appear clearly when using synthetic analogs [65], many studies have used them as an exogenous application to help plants under different stress conditions [17,41,45,68,77].

Strigolactones (SLs) are generated from carotenoids and are regarded as secondary metabolites, which mainly have roles in shoot branching and modified plants phenotypic characteristics to attenuate the effect of gravitropism phenomenon. [84,85]. Analogs of SLs are intended to have the same bioactivity as SLs found in nature. SL analogs’ synthesis is explained, along with information on how stable they are in aqueous solutions [86]. There are many structures of typical SL analogs such as GR24, Nijmegen-1, and EM1 (derived from ethyl 2-phenylacetate). SLs analogs are designed to have the same bioactivity as natural SLs. The synthesis of these SL analogs is reported together with the stability in an aqueous solution [86]. The analogs are different in their functions and characteristics. For instance, Nijmegen-1 hydrolyzes at a faster rate than GR24. In field trials with Nijmegen-1, it was established that the correct formulation prevents early hydrolysis, and the suicidal germination technique is still feasible [86,87,88,89]. In the same trend, many other SL analogs have different characteristics and effects on plant growth and development. Exogenous synthetic analogs, such as GR5, GR7, and GR24, (considered a reference of strigolactone), have been applied to analyze the role of strigolactone in different plant organs and physiological systems [90]. The use of GR24 under dark settings reduced cytoskeletal rearrangement, revealing a new mechanistic link between cytoskeletal behavior and strigolactone signaling light sensitivity [65]. Although GR24 is considered a reference of strigolactone and has been used experimentally on various crops, GR5 and GR7 have been used for their simple structure. Furthermore, other analogs with EGO and ST groups have been used for their different functions. CISA-1 is a fluorescent strigolactone molecule that has been synthesized via a novel method by Rasmussen et al. [91]. Karrikins are another alternative analog with different hormonal effects [15]. Further information is needed to clarify the different characteristics and roles of the huge number of developed SLs analogs. 

## 5. SLs Are a Messenger of Other Microorganisms

### 5.1. Soil and Rhizosphere Microbiome in Relations to Abiotic Stress

Plants interact and communicate with a wide range of microbes, insects, birds, and other plant species above and below soil [81,92]. Soil contains nematodes, fungi, bacteria, invertebrates, and roots of neighboring or parasitic plants [85,93,94,95,96]. The rhizosphere is a narrow layer of soil surrounding the roots but includes billions of microbial cells per gram of root. Soil microorganisms are the greatest microbial community, which includes a great variation of microbes [97]. 

Abiotic stress moderates various metabolites to enhance changes in root exudates, which increases the microbial abundance to alleviate stress [98]. The plant goes through the ‘asking for help’ strategy by exudate messenger substances that attract the beneficial microorganisms, which could penetrate the endosphere or live in the rhizosphere [98]. Strigolactone is considered one of these exudate substances that attracts the rhizosphere microorganisms. For instance, when the plant is exposed to nitrogen (N) deficiency, leguminous plants release larger amounts of flavonoids to attract N-fixing bacteria [98]. There is a relationship between the rhizosphere microbiome and nutrients, especially phosphorus and nitrogen absorption and uptake. In the soil microbiome, there are identified microorganisms that improve the availability of phosphorus and nitrogen to enhance absorption processes [99]. The rhizosphere affects plant health not only in nutrient uptake, but also in the prevention of pathogen colonization, and enhancement of plant immunity toward insect attack [97]. Many other phenolic compounds are also important to enhance plant tolerant and attract a relative abundance of microbial taxa. The harmony of metabolites, gene transcription, and the rhizosphere microbiome is a trend approach that includes the metabolomic, transcriptomic, and metagenomic studies [98] (Figure 3). 

A number of soil microorganisms have developed strategies to adapt against variable environmental conditions. They construct the soil organic carbon (SOC), which in turn enhances the soil water holding capacity [100]. Increasing the water holding capacity of soil is a physical strategy against the water scarcity. Many considerations are related to environment-microorganism relations. Physiological state, gene transcription, translation, mutations, and more are taken into consideration by microorganisms in order to face abiotic stress and support plants (Figure 3). SLs act as lighthouses to these microorganisms. SLs are the key factors to attracting numerous soil microorganisms and adjusting the soil ecosystem. To promote a network of mutualistically beneficial relations with these microorganisms [101,102]. Together, SLs and soil microorganisms modify the soil chemical composition. The root exudate SLs into the soil attract and recruit microorganisms [103]. The combination of SLs and these microorganisms promote tolerance mechanisms that contribute to terminating the present stress on the plant.

Moreover, Soil microbiome diversity is altered depending on changes in the surrounding ecology including microbial communities [100]. For instance, the high temperature affects the soil microbial communities by changing the spread of species or changing a single species itself [100]. Different scenarios describe the changing of the microbiome according to the climatic change’s stresses. Different soil ecosystems have different impacts on abiotic stress. Physical, chemical and biological factors play basic roles in the microbial ecosystem. Soil pH, salinity, electrical conductivity, soil texture, and other features affect the soil ecosystem as well [100]. SLs are a generator of soil microbial diversity [101,103] (Figure 2 and Figure 3). This changes in the diversity affects the soil and plant relations to improve the plant tolerance, such as improving the soil organic matter which is a magnificent media for nutrient and water holding [104]. This cooperation gives benefits to both domains of creatures to survive against the abiotic stress. 

### 5.2. Phyllosphere Microbiome and Their Relations to Abiotic Stress

The Phyllosphere microbiome includes the microorganisms located at the above-ground plant parts such as stem, leaf, flowers, and fruit surface. Phyllosphere is divided into two parts which are phylloplane and phyllotelma, which includes the plant endosphere [105,106]. The phyllosphere has a wide range of environments. The different harbors of the phyllosphere diversify microorganism communities. This diversity influences ecosystem functioning through enhancing the plant’s immune responses and defense mechanisms toward the different abiotic stresses. The diversity occurs by modifying soil microbial community to allow definite phylotypes to colonialize the plants [105]. For example, *lactobacillus* spp. which is resides in/on pollens play a role in pollination processes. In addition to phyllosphere microorganisms’ relation to the plants, they have microbe-microbe interactions [105] (Figure 3). It is revealed that SLs have impact on the leaf’s bacteria and fungi and on the microbial interactions with the aerial tissues, probably affecting the actions of microorganisms on phyllosphere [103]. It is possible that SLs may indirectly affect the assembly of the phyllosphere microbiome through its crosstalk with other hormones such as cytokinin which has a significant role in this [107]. There is communication between the shoot and roots microbiome. For instance, when leaves are infected by a pathogen, the root attracts beneficial microorganisms from the rhizosphere [108], in addition to many methods of communication of the two microbial communities. Many substances have roles in this communication as flavonoids [108]. SLs are the most important attraction factor, as it well known. SLs modify some metabolic pathways including flavonoids [109]. 

The plant genotype is a necessary element in determining the bacterial composition of the phyllosphere. This appeared in a study comparing the phyllosphere communities between different plant species such as pine and tropical trees. The abundance could be higher or lower depending on the genotype of the plant species. Plants can use the phyllosphere microbiome in different strategies by using secondary metabolites, gene expression, and protein upregulating during stress [106] (Figure 3). SLs have a key role in secondary metabolite interactions with different enzymatic reactions. This group of carlactonic compounds play a great role in the gene expression. Plant genotypes determine the type of strigolactone present so the exudate strigolactone composition is specific to the particular plant. Thus, this changes the microbiome combination. 

Phyllosphere microbial colonizers communicate with plants to face different abiotic stresses. While the reactive oxygen species (ROS) damage proteins, lipids, and nucleic acids under stress, the microorganisms work against the stress to reduce the damage of ROS to enhance physiological processes such as photosynthesis This is a complex process that mainly occurs by stimuli definite enzyme production to alleviate ROS and other metabolites that are harmful under stress. These processes have a vital role in the protection of DNA by activating the DNA repair mechanisms. The accumulation of microbial communities on the leaf surface protects the plant from dehydration under heat stress with various considerations to the humidity conditions [106]. The leaf microbiome uses aggregate formation and extracellular polymeric substances (EPS) to resist desiccation [110] (Figure 3).

These microorganisms support and improve plant growth and development under several environmental conditions, such as temperature, radiation, water content, and nutrient that impact the phyllosphere microbial community [110]. Water is a vital limiting factor of leaf survivability. Plants exudate different substances from the leaf cells such as amino acids which provide the phyllosphere microorganisms with nitrogen, and metabolites support the growth of the microbes [110]. These complex relations between water, nutrients, and organic substances support both plants and microorganisms to survive and grow [110] (Figure 3).

The combination of the phyllosphere microbiome and cell chemical exudates explain that SLs might have direct or indirect impact on the microbiome. The cross talk between SLs, other hormones and other metabolites may enhance the phyllosphere microbiome. There is much evidence that different cellular chemical substances affect this microbial community [111]. The relationship between SLs and Phyllosphere microbiome is not completely clear and needs further experimental work to identify the cooperations between them.

### 5.3. SLs Are Messengers in Microorganisms and Plant Crosstalk 

Recent research has discovered that SLs act as messengers in the crosstalk occurring among the soil, rhizosphere, endosphere microorganisms, and root system cells. They not only work as hormone messengers but also as interaction messengers and signaling regulator. The symbiotic relationship between the rhizosphere and microorganisms is important for plant health, these relationships are more important in soil rich with organic matter and soil microorganisms [112,113]. Some of these microbes have symbiotic, parasitic, or other living relationships. To enhance plant growth and health, it is essential to investigate the abundance, diversity, and function of microorganisms in the rhizosphere microbiome [112,113]. The synthetic analog GR24 was used to investigate SL regulation in integrating plants with the subsurface ecosystem. Different biotic and abiotic stressors can be inhibited by the plant’s surrounding microorganisms, which is one of the plant’s main stress-coping systems. Rhizosphere microorganisms can assist plants in absorbing nutrients from the soil. An additional function of the microorganisms is to modify soil pH and secrete organic matter or Osmo-regulators into the soil, which act to buffer and condition the soil [114] (Figure 3). 

One of the most important beneficial microorganisms in the soil and rhizosphere is the Arbuscular mycorrhizal fungi (AMF). The relationship between plants and this fungus is an ideal example of a symbiotic relationship. The symbiotic association between AMF and plant roots promotes nutrient uptake, enhanced tolerance against pests, disease, drought stress, heavy metal residual, and improved soil structure [115]. It was reported that *Arabidopsis*, *Orobanche*, and AM fungi possess variations in receptor sensitivity to SL analogs, probably due to variations in SL receptors among the different species [65]. SLs are rhizosphere signaling compounds that mediate host location in arbuscular mycorrhizal (AM) fungi and parasitic plants such as *Orbanche* [7,116,117]. Phosphate availability enhances chemical signaling and performs dual functions in attracting beneficial AM fungus and damaging parasitic plants [27,118]. Previous researchers cited the relationship between AMF, plant roots, and rhizosphere. AM fungi have multi-function relations with roots in the rhizosphere. SLs strengthen the symbiotic association between roots and AMF under different stress conditions and phosphorus deficiency [29,31,119,120] (Figure 3).

Both rhizosphere and phyllosphere ecosystems of plants have complex relations with each other and within their microbial communities. SLs are moderators between plants and microbes in addition to their hormonal and metabolic roles [81,121,122]. SLs appear to attract microorganisms that can help plants cope with biotic and abiotic stresses. Nodulation, colonization, and symbiosis are ways that bacteria and their plant hosts form a symbiotic relationships [123]. The formation of these relationships is influenced by the root secretions, and the plants can identify the composition of their microbiome by these active secretions. In some plants’ rhizosphere it is found that organic acids such as citric, succinic, and malic acids are the most common secretions. It is also determined that many of these bacteria strains need organic acids as the only carbon source to grow in vitro, which emphasizes the vital role of root secretion for bacterial survival. In addition, it reflects the colonizing ability and crosstalk relations between plants and the rhizobacterial strains [97]. Many bacteria associated with the plants may produce and utilize N-acyl-homoserine lactones (AHLs). It is used not only in signaling and communication with each other but also in their gene expression. Cell-to-cell communication is called ‘ quorum sensing’ (QS) [97] (Figure 3). 

Strigolactones are root exudation substances which are related to the microbial association. However, the understanding of the communication between strigolactones and microorganisms to face abiotic stress is not clear. There are many studies attempting to explain approaches to this association [121]. Supportive evidence demonstrated the role of strigolactones in the attraction and signaling of microorganisms to enhance tolerance against nutrition deficiency, drought, salinity and light stress to protect plants from these abiotic stresses [16,45,66,121] (Figure 2 and Figure 3). 

## 6. Strigolactones Alter Vegetative Growth during Abiotic Stress

### 6.1. Effects of Strigolactones on Shoot Branching and Bud Outgrowth 

Strigolactone has been identified as a branching reduction factor [7,117]. SLs inhibit auxin stimulation; when SLs are found in the cell, they inhibit the influence of auxin activities and inhibit branching as mentioned in Figure 2. Decapitation-induced bud outgrowth was reported on shoot branching in the late 1930s [124]. The branching reductions relate to various factors including stimulation compounds in pathways. Brewer et al., [68] reported that the application of GR24, a synthetic analog of strigolactone, towards the axillary buds of wild-type peas affected the bud outgrowth and consequently also reduced branch initiation. In addition, lateral bud growth was observed in untreated plants. While N-1-naphthylphthalamic acid (NPA) has a great response to the bud’s outgrowth, it harmonizes the effects among auxin, SLs, and cytokinins toward organizing the bud’s outgrowth and inhibition [68]. The regulation of shoot branching is one of the prominent effects of SLs in plants. Furthermore, strigolactone treatment to small buds of mutant or decapitated pea plants quickly inhabited outgrowth, however, N-1-naphthylphthalamic acid (NPA), an auxin transport inhibitor, took several days to restrict growth severely. Inhibiting bud growth for shoot branching against apical dominance includes suppressing growth by other growing buds or shoots. It is thought that the flow of auxin in stems and buds is involved in these processes [67]. Moreover, in peas (*Pisum sativum*) *RAMOSUS* (*RMS*), branching genes control the synthesis and perception of a long-distance inhibitory branching signal produced in the stem; roots were affected by the SLs and auxin as well.

Additionally, stem girdling prevents auxin transport, and GR24 suppresses lateral bud expansion in plants [67]. The strigolactones consequently affect the dry matter and water content in plant organs [45,77]. D53-like SMXLs regulate shoot branching in Arabidopsis [39], meanwhile, some studies reported that even the application of SLs on different sizes of buds has the same branching inhibition response] [17]. The apical dominance and bud outgrowth relate to the hormonal effects of not only auxin (IAA) but also cytokinin (CK) through several pathway steps. Strigolactone acts as a stimulation factor in the apical dominance hormone signaling and biosynthesis pathways to start the bud outgrowth promoting or inhibiting process [67]. *IPT1* and *IPT2* genes play a key role in regulating bud outgrowth under stem girdling or decapitation. They work with *RMS1* and *RMS5* genes, which are certainly related to IAA and CK hormones with their relations to SLs [42,67]. 

### 6.2. Stomatal Responses

Stomatal openings, guarded by a pair of specialized guard cells in the leaf epidermis, are referred to as leaf windows that connect plants to their external environment (Figure 2). Stomatal openings protect plants from various stressors such as drought and high temperatures. GR24 affects the role of ABA on stomatal closure [17]. Moreover, the guard cells of stomata act as a mechanism for defense as they provide the plant with protection from pathogens. In general, GR24 has been used as a positive control to research the biological activity of SLs, and it has been discovered that SLs regulate abiotic stressors and play distinct roles in stress responses (Figure 2).

## 7. SLs Regulates Root Morphogenesis

SLs influence root growth including main root length, lateral root development, and root hair elongation. According to previous reports, SLs have a role in auxin transport, either locally in the root or systemically from the shoot [42,56,68,69,70,74,125,126]. The Rhizosphere plays an essential role in nutrient and water absorption with the help of microorganisms, which improve the plant’s capacity to cope with abiotic challenges including drought, salinity, as well as to high and/or low temperatures [65,84,93]. In addition, rhizosphere microorganisms are a key factor in improving root growth and development. The interactions between the host plant and its associated microbiome are harmonized by chemical responses. Mainly, chemical signaling between the plant and microbiome in rhizospheres occurs by sending an exudation of secondary metabolites including SLs [93] (Figure 2 and Figure 3).

Root hairs are tiny root structures that allow plants to absorb water and nutrients. Morphological modifications such as root hair initiation, elongation, or cellular alterations are required to carry out physiological developments. The root apical meristem is a crucial regulatory tissue for root hair development and may be influenced physiologically. The initiation and development of root hair were both affected by SLs [55,65]. Plants continuously extend their root and shoot systems through the action of meristems at their growing tips. By regulating their meristems which are active, plants adjust their body plans to suit local environmental conditions [23,58]. SLs promote primary root hair initiation and elongation and root fresh weight, facilitating the absorption of water and increasing the number of roots [127]. 

Ruyter-Spira et al. [126] studied the impact of GR24 on root architecture and found that using GR24 as SLs analogs leads to an increase in the primary root length (PRL) while GR24 suppresses lateral root density (LRD) by suppressing lateral root (LR) outgrowth. GR24-induced suppression of Lateral root primordia (LRP) outgrowth is partially mediated through decreased shoot-derived auxin levels, while endogenous SLs stimulate LR outgrowth during Pi-Limiting conditions.

Consequently, the plant modifies the root architecture to absorb water and nutrients under the abiotic stress. Roots exudate SLs to improve their structure with the support of the rhizosphere microbiome The application of SLs in the form of one of its’ analogs may improve the root architecture to implement the same functions. 

## 8. Photosynthesis Improving under Abiotic Stress 

Photosynthesis is an extremely vital biochemical and metabolic process in plants. SLs alleviate plant stress by improving photosynthesis and reducing oxidative stress [128,129,130]. Light is the main critical factor for photosynthesis and chlorophyll is the sensor regulator of photosynthesis [45]. Simultaneously, SLs enhance the content of chlorophyll, thus enhancing the photosynthesis process [45,77,131]. For instance, SLs boosted photosynthesis under low or normal light stress with the application of GR24 in tomato plants, which increased the chlorophyll content, net photosynthetic rate, photochemical efficiency of the photosystem, and the effective quantum yield of PSII [45].

## 9. Some Mechanisms against Different Stresses

### 9.1. SLs Enhance Phosphorus and Nitrogen Uptaking under Starvation Stress

The relationship between soil macronutrients and SL production in the root system has been reported in various plant species. This relation is an essential regulatory function of the SLs in nutrient deficiency. There are many scenarios of this effect, some of which revealed the microorganism and its role in attracting them. One of these scenarios is related to arbuscular mycorrhiza fungi (AMF) which are one of the most important microorganisms because it mediates the interaction among plant, the rhizosphere, the soil, and the entire ecosystem. AMF is an effective moderator of nutrient uptake. AMF is highly affected by SL concentration in the root system. This may explain the extent of AMF penetration, especially as the plant faces phosphorus deficiency [29,31,119,120].

Nitrogen (N), phosphorus (P), and potassium (K) are essential macronutrients, known as NPK. NPK are vital elements for growth and development in plants [127] and are associated with necessary regulatory functions, chemical compositions, and accumulation of secondary metabolites [132]. Aside from exhibiting crosstalk with the soil, the SLs are modulators and regulators of N and P reduction in the rhizosphere. Many studies reported the relationship among N, P, and SLs in the context of nutrient deficiency and signaling mechanisms [24,25,50]. Phosphorus participates in various morphological functions such as primary root growth and the entire root system architecture (Figure 2 and Figure 3). The availability of phosphorus and nitrogen in the root zone is one of the major regulators of SL production. The interaction between SLs and nutrient-related bacteria, such as AM fungus and other nodulation microorganisms plays an important role in preventing the plant from running out of N and P (Figure 2 and Figure 3) [23,24]. 

Phosphorus is present in the form of inorganic phosphate (Pi) in soil, which has poor mobility as well as low availability in the rhizosphere [25]. It is involved in various metabolic and physiological activities in plants including energy transfer, photosynthesis, the transformation of sugars and starches, nutrient movement within the plant, and the transfer of genetic characteristics from one generation to the next. Additionally, phosphorus contributes to the structure of genetic components including DNA, RNA, ATP, and membrane phospholipids. Phosphorus has a substantial impact on several metabolic processes and activities in plants, including phosphorylation reactions. SL application to the roots at a trace concentration alleviated the Pi deficiency [26,27]. 

The root branching under phosphorus deficiency and 29-epi-5-deoxystrigol (epi-5DS) levels in roots are associated with the number of tillers outgrowth [26,133]. The weight of roots and shoots is similarly affected by phosphorus deficiency. Ref. [27] studied the effect of SLs in tomato (*Solanum Lycopersicum*) under phosphorus deficiency. The results showed that tomato root induced the hyphal branching of AM fungi with the physiological combination of abscisic acid (ABA) in the presence of the carotenoid biosynthesis inhibitor fluridone. It was also found that phosphate deficiency distinctly increased while fluridone concentrations significantly decreased. 

SLs have a pivotal role in nutrient allocation and the mechanisms of resistance against nutrient starvation [134]. The regulation of shoot/root architecture, the promotion of contact between roots and fungus or bacteria, as well as the influence on the germination of parasitic plants, are a few of the signaling pathways connected to SLs. The production and exudation of SLs are enhanced in the presence of phosphorus and nitrogen shortage in roots. SLs may play a role in the complex response to nutritional stress which involves plant metabolic activities and is associated with other microorganisms to reduce the stress impacts on plants [23,131]. Different morphological changes in the shoot are a result of the anatomical and physiological changes (phenotypical changes) that have occurred because of the relationship during phosphorus starvation. These physiological changes include leaf expansion, specific leaf area (SLA), specific root architecture [127], shoot growth, as well as blade and sheath characteristics [28,57]. Ref. [50] found that plants prioritize N over P status by affecting SL biosynthesis. 

### 9.2. Effect of SLs on Drought

The biological activity of SLs in response to drought has been studied using the positive control GR24, and it has been found that SLs regulate abiotic stressors and play different roles in stress response (Figure 2 and Figure 3). SL-depleted plants are hypersensitive to drought owing to stomatal hyposensitivity to abscisic acid and contribute to drought acclimation in shoots. However, under drought, SL accumulation is suppressed in the roots suggesting that their metabolic activities and functions are organ-specific [135]. These adverse effects might be physiological such as a decrease in photosynthetic rate, respiration, osmotic imbalance, or membrane system damage. Under drought conditions, SLs enhance the photosynthesis process, which can positively impact a variety of physiological systems. SLs may influence ribosome-mediated carbon metabolism, starch and sucrose metabolism, flavonoid production, and circadian rhythm. There are connections between the SLs and the activation of different genes such as antioxidant enzyme genes [77]. Stomatal conductance, morphological changes and the modification of epidermis cells are some of the significant changes in plants caused by drought stress [17]. In grapevines, SLs can exogenously reduce drought symptoms [77]. However, there is a lack of knowledge on transcription levels regarding drought stress mechanisms in citrus. It is evident that the SL exogenous application reduces drought stress in grapes by increasing relative water content and decreasing electrical conductivity, in addition to reducing drought impact.

The relationship among SLs and stomatal conductance, electron transport, and leaf water potential as an inducing factor during drought may influence chlorophyll content. SLs may also influence chlorophyll under drought stress. The application of exogenous plant growth regulators has an alleviative effect on drought stress signs. Min et al. [17] studied the effects of SLs on grape seedlings under drought stress and found that the plants treated with GR24 showed higher drought tolerance in terms of decreased electrolyte leakage, fewer open stomata, and less ROS, as well as increased relative water content, chlorophyll content, photosynthetic rate, and malondialdehyde (MDA) content. The exogenous application of GR24 can alleviate the adverse effects of drought on stomatal closure since it regulates stomatal closure via ABA or ROS (Figure 2, Figure 3 and Figure 4), regulates chlorophyll components and photosynthesis, and activates antioxidant defense ability [17,77]. Several drought-tolerant genes were reported including antioxidase genes *CAT1*, *GSHPX1*, *GSHPX2*, *DCP42*, and *APX6* transcription factors *NAC*, *WRKY*, *MYB*, and *D14*; all of which are key genes involved in the signal transduction of SLs in grapes [77]. Meanwhile, Cytokinins (CKs), ABA, and SLs act as negative and positive regulators in-plant drought responses. There is organized crosstalk between CK, ABA, and SLs signaling pathways in many mechanisms underlying plant drought acclimatization [4].

### 9.3. Senescence as a Defense Mechanism

Senescence is the ultimate physiological state of plant organ development during which all cells break down, redistribute, and relocate their nutrients among different organs [83,136,137,138]. Hence, senescence aids plants in coping with a variety of environmental stresses [15,139]. Deciduous trees shed their leaves as a physiological senescence process, occurring seasonally, to face the winter chilling conditions and dormancy. Leaf senescence is regulated by various factors such as temperature, light intensity, stress, aging, and concentration of phytohormones [83]. SLs affect leaf senescence as *ore9*/*max2* exhibits a delayed senescence phenotype. Ueda et al. [83] investigated the role of SLs in leaf senescence and found that SLs exhibited a delayed senescence phenomenon, indicating that SLs are involved in leaf senescence regulation (Figure 2). Furthermore, Ueda et al. [83] studied the relationship among SLs, phosphorus deficiency, and senescence, and found that GR24 application accelerated leaf senescence in the entire SL-deficient mutants under phosphate deficiency and dark-induced leaf senescence. The effects of GR24 appearing in *d10* compared to *d17* mutant genes suggested that SLs, as a response to phosphate deficiency, regulate leaf senescence [138]. The complex analysis mentioned the correlation between ethylene biosynthesis and photosynthesis. Numerous ethylene biosynthesis and genes responsible were up-regulated under both IAA and IAA+GR24 treatments, suggesting their involvement in the ethylene biosynthesis regulation [56].

## 10. SLs and Surrounding Eco-System Organisms

SLs are communicators to the surrounding environment and the other organisms in that ecosystem. For instance, they are involved as messengers for parasitic weeds, root-hair elongation, regulating plant development, seed germination, and inhibition [65,118]. Initially, the primary role of SLs was reported on parasitic weed seed germination, growth and development, and growth habitats. Strigol, a member of the SL family, has been identified as a germination influencer for the parasitic plants in *Striga* spp. [93]. Studying signaling studied pathways revealed the inhibition and stimulation effects of SLs on parasitic weeds, where receptors such as *KAI2/HTL* was reported to be involved in this pathway [93]. SLs and SL-like compounds not only attract parasitic weeds but also attract beneficial soil microbes [18]. The roots exudate SLs which activate the germination of the parasitic weed [74,109]. They were involved in plant adaptation as well as resource competition with surrounding organisms. Moss is the best example of a plant utilizing SLs to respond to its surrounding environment [40].

Witchweeds (*Striga* spp.) and broomrapes (*Orobanche* and *Phelipanche* spp.) belong to the family Orobanchaceae and are the two most destructive root parasitic plants. They are causing extraordinary losses in cultivated crops around the world [40]. Seeds from these root parasites germinate when exposed to a chemical stimulus. Germination stimulants’ exuding from plant roots, therefore, SLs have been considered the common germination stimulants.

## 11. Conclusions and Prospects

SLs are future promising growth regulators which have many regulatory functions in various plant tissues with multiple physiological and genetic influences. They have significant network processes inside the plant organs, as well as with other organisms in the ecosystem. Microorganisms are the main partner to SLs in many biological processes in plants. As plants and their products are under the menace of global climate change aspects, SLs could be a useful tool in helping plant development and productivity under biotic and abiotic stress. The improvement of plant growth under stress will support sustainability under the circumstances of climatic changes. However, since phyllosphere microorganisms play a role in above-ground plant development, investigation of their roles is still needed. The relationship among soil, rhizosphere, and plant roots needs SLs as connecting agent. That support the concept of ‘asking for help’. Soil and plant microbiomes are extremely valuable to plants’ wellbeing. Keeping in mind the importance of SLs in relation to microbes, there is a need to investigate the effect of SLs on various plant species, especially fruit trees. Additionally, the role of SLs on some plant phenotypes is still considered a research gap. Organic and sustainable use of derivatives of lactones as a phytohormone in organic, sustainable, and biodynamic farming needs to be studied under the necessity to investigate the effect of extracted natural SLs on the plant crops in relation to environmental aspects.

## Data Availability

All data is contained in this manuscript.
